# Temperature and pressure adaptation of a sulfate reducer from the deep subsurface

**DOI:** 10.3389/fmicb.2015.01078

**Published:** 2015-10-06

**Authors:** Katja Fichtel, Jörn Logemann, Jörg Fichtel, Jürgen Rullkötter, Heribert Cypionka, Bert Engelen

**Affiliations:** ^1^Paleomicrobiology Group, Institute for Chemistry and Biology of the Marine Environment, University of Oldenburg, OldenburgGermany; ^2^Organic Geochemistry Group, Institute for Chemistry and Biology of the Marine Environment, University of Oldenburg, OldenburgGermany

**Keywords:** *Desulfovibrio*, fatty acids, intact polar lipids, Juan de Fuca Ridge, ornithine, phospholipids

## Abstract

Microbial life in deep marine subsurface faces increasing temperatures and hydrostatic pressure with depth. In this study, we have examined growth characteristics and temperature-related adaptation of the *Desulfovibrio indonesiensis* strain P23 to the *in situ* pressure of 30 MPa. The strain originates from the deep subsurface of the eastern flank of the Juan de Fuca Ridge (IODP Site U1301). The organism was isolated at 20°C and atmospheric pressure from ~61°C-warm sediments approximately 5 m above the sediment–basement interface. In comparison to standard laboratory conditions (20°C and 0.1 MPa), faster growth was recorded when incubated at *in situ* pressure and high temperature (45°C), while cell filamentation was induced by further compression. The maximum growth temperature shifted from 48°C at atmospheric pressure to 50°C under high-pressure conditions. Complementary cellular lipid analyses revealed a two-step response of membrane viscosity to increasing temperature with an exchange of unsaturated by saturated fatty acids and subsequent change from branched to unbranched alkyl moieties. While temperature had a stronger effect on the degree of fatty acid saturation and restructuring of main phospholipids, pressure mainly affected branching and length of side chains. The simultaneous decrease of temperature and pressure to ambient laboratory conditions allowed the cultivation of our moderately thermophilic strain. This may in turn be one key to a successful isolation of microorganisms from the deep subsurface adapted to high temperature and pressure.

## Introduction

The volume of world’s oceans 200 m below sea level constitutes more than 95% of all aquatic habitats (Michiels et al., 2008). Additionally, the subseafloor represents a large reservoir for prokaryotic life (Whitman et al., 1998; Kallmeyer et al., 2012) and even extends into the upper oceanic crust (Heberling et al., 2010; Edwards et al., 2011; Orcutt et al., 2011).

Most studies to identify the microbial diversity within the deep marine subsurface are based on cultivation-independent approaches (Marchesi et al., 2001; Kormas et al., 2003; Inagaki et al., 2006; Webster et al., 2006; Biddle et al., 2008). Cultivation attempts are increasing (Bale et al., 1997; Wellsbury et al., 2002; Batzke et al., 2007; Kobayashi et al., 2008; Miyazaki et al., 2012; Takai et al., 2013), but are still the exception. Even though novel high-throughput techniques such as metagenomics or single cell genomics are important for predicting *in situ* ecological functions (Teske, 2006; Lauro and Bartlett, 2008), the isolation of microorganisms from deep ecosystems is seen as the “gold standard” to identify putative physiological capabilities and specific adaptation mechanisms (Giovannoni and Stingl, 2007) to subseafloor habitats. Thus, isolates are still indispensable to verify metabolic pathways that are only detected by *in silico* analysis.

One interesting aspect to be analyzed on deep subsurface microorganisms is their ability to live under elevated hydrostatic pressure. Taking the deep ocean, the marine subsurface and the oceanic crust into account, the majority of all prokaryotic cells in the environment are facing high-pressure conditions. High hydrostatic pressure has ‘pervasive effects’ (Eloe et al., 2011) on microbial physiology, influencing macromolecular structures or cellular processes such as metabolisms, cell growth, viability, and motility (Bartlett, 2002; Abe, 2007). Previous investigations of pressure adaptation of marine microorganisms were performed mainly on psychrophilic deep-sea bacteria (DeLong and Yayanos, 1985; Wirsen et al., 1986; Kato et al., 2008) and some data exist for thermophilic bacteria and hyperthermophilic archaea from hydrothermal vents (Jannasch et al., 1992; Alain et al., 2002). Physiological data on mesophilic piezophiles were limited to a few isolates (Kaneko et al., 2000; Alazard et al., 2003; Khelaifia et al., 2011). Recent studies on these bacteria include isolates affiliated to *Shewanella profunda* (Toffin et al., 2004; Picard et al., 2015), *Shewanella piezotolerans* (Xiao et al., 2007; Wang et al., 2009; Wu et al., 2013), *Desulfovibrio hydrothermalis* (Amrani et al., 2014), and *Desulfovibrio piezophilus* (Khelaifia et al., 2011; Pradel et al., 2013). Moreover, pressure studies on isolates from marine subsurface sediments are rare (Bale et al., 1997; Barnes et al., 1998; Mangelsdorf et al., 2005; Toffin et al., 2005). Generally, the adaptation capacity depends on the ability of microorganisms to regulate structure and organization of their cell membrane as a response to changes in temperature and pressure in order to maintain the membrane fluidity necessary for sustaining biological functions (‘homeoviscous adaptation’, see Sinensky, 1974; Macdonald, 1988; Somero, 1992; Kaye and Baross, 2004). The reorganization of membrane constituents and proteins influences the membrane lipid composition, the degree of saturation of membrane-bound fatty acids (FAs), as well as their chain length and branching (DeLong and Yayanos, 1985, 1986; Wirsen et al., 1986; Yano et al., 1998; Mangelsdorf et al., 2005).

In our previous study, we have isolated several sulfate-reducing bacteria from subsurface sediments of the Juan de Fuca Ridge (Fichtel et al., 2012). The isolates were obtained from depths down to 260 m below the seafloor, about 5 m above the sediment–basement interface. The sampling location (IODP Site U1301) was target of several microbiological investigations of the sediments (Lever et al., 2010) but much more of the oceanic crust below (Jungbluth et al., 2014; Robador et al., 2015). IODP site U1301 exhibited a water depth of 2656 m, corresponding to an *in situ* pressure of ~30 MPa for the deepest sediments analyzed. At this site, highly compacted hemipelagic clay with a particle size of <2 μm and a bulk density of ~2 g/cm^3^ covers the basaltic crust (Zühlsdorff et al., 2005). The sediments serve as a hydrogeologic barrier for advective fluid-flow from the basaltic aquifer into the sediments and do not allow any transportation of larger particles such as microbial cells. On the other hand, low-temperature hydrothermal fluids diffuse from the underlying oceanic crust into the sediment column, resulting in a steep temperature gradient of 2 to 62°C from the ocean floor to the basement. These crustal fluids provide energy sources like sulfate (16 mM) from below, thus stimulating sulfate-reducing communities to thrive within this habitat (Engelen et al., 2008).

Pure cultures were obtained under standard laboratory conditions, i.e., at atmospheric pressure and 20°C. The sulfate-reducing bacteria isolated from the deepest sediments above the basement solely belonged to the Deltaproteobacteria, namely one *Desulfotignum balticum*-affiliated strain from 260 m below seafloor (mbsf) and three strains related to *Desulfovibrio indonesiensis* from 240, 252, and 260 mbsf. As members of the *Deltaproteobacteria* are not known for forming resting stages, they are presumed to belong to active microbial populations of the deep subsurface. Physiological characterization of the isolates revealed that the *D. indonesiensis*-affiliated strains have a temperature range of growth from 10 to 48°C, and exhibit both, a chemoheterotrophic and lithoautotrophic lifestyle (Fichtel et al., 2012). Interestingly, the temperature range of growth did not reach *in situ* temperatures of 56–61°C. As temperature and pressure can have opposing influence on the cell membrane, an insufficient combination of both parameters may result in an inhibition of cross-membrane processes or even the disintegration of cells (Mangelsdorf et al., 2005). Thus, the question arose whether incubation under *in situ* pressure would induce a shift in their temperature range of growth.

Six sulfate-reducing isolates from deep subsurface sediments of IODP Site U1301 were examined for growth under high pressure and various temperatures. For the present study, we chose *D. indonesiensis* strain P23 as a representative to be analyzed in more detail. The isolate derived from the deepest sediment sample and exhibited relatively fast growth both, under high hydrostatic pressure and high temperatures. Microbiological investigations such as microscopic analyses and measurements of growth rates at different combinations of temperatures and pressures were complemented by membrane-lipid analysis to identify a cellular response to changing incubation conditions.

## Materials and Methods

### Bacterial Strains, their Origin and Growth Conditions

Pure cultures of strictly anaerobic, sulfate-reducing bacteria used in this study were obtained from up to 260 m deep subseafloor sediments. Samples were collected in the northeast Pacific at the Eastern Flank of the Juan de Fuca Ridge, IODP Site U1301 (47°45.28′N, 127°45.80′W; water depth: 2656 m) during IODP Expedition 301 in 2004. Details on environmental conditions, sampling, and contamination tests have been reported previously (Expedition 301 Scientists, 2005; Lever et al., 2006; Engelen et al., 2008; Fichtel et al., 2012). Enrichment and isolation of pure cultures were performed at ambient conditions, i.e., atmospheric pressure of ~0.1 MPa and 20°C. Cultivation procedures and phylogenetic analyses of cultures obtained are given in Fichtel et al. (2012). Strain P23, affiliated to *D. indonesiensis* (99% 16S rRNA gene sequence similarity), was analyzed representatively for pressure and temperature adaptation in more detail.

Five additional isolates from the same sampling site (*D. indonesiensis* strain P12 from 252 mbsf, *D. indonesiensis* strain P34 from 240 mbsf, *D. aespoeensis* strain P20 from 1.3 mbsf, *Desulfotignum balticum* strain P18 from 260 mbsf, *Desulfosporosinus lacus* strain P26 from 1.3 mbsf), and the type strain of *D. indonesiensis* (Ind1^T^, DSM 1512) were analyzed in less detail for comparison. All strains were pre-cultured to early stationary phase at atmospheric pressure and 25 to 35°C in sulfate-containing (28 mM) artificial seawater media that had originally been used for isolation (Fichtel et al., 2012). Lactate (10 mM) or betaine (5 mM) was used as carbon source. Growth was routinely followed by photometrical determination of sulfide in form of colloidal CuS at 480 nm (Cord-Ruwisch, 1985) and of cell protein concentrations at 595 nm after Bradford (1976) as well as by visual inspection of the cells using phase-contrast microscopy. Transmission electron microscopy (TEM) of strain P12 was performed with air-dried, unfixed cells as described by Fichtel et al. (2012).

### General Setup for Pressure Incubations

All pure cultures were examined as to whether they were able to grow under pressure (10 to 40 MPa). Bacterial growth experiments were performed in ‘high-pressure steel vessels’ [High Pressure Equipment (HiP) Company, Linden, PA, USA]. Inoculations were done in 60 or 70 ml serum bottles containing freshly prepared culture media and sealed with rubber stopper and crimp caps. Pre-cultures (4% of final volume) were injected and bottles were completely filled with the respective media avoiding any gas bubbles. Three serum bottles were placed inside a pre-heated pressure vessel filled with distilled water. Samples were set under hydrostatic pressure by means of a hand operated ‘high-pressure generator’ (model 81-5.75-10, HiP) using distilled water as hydraulic fluid. For subsampling, the vessel was carefully depressurized (~1 min). The bottles were subsampled (5–6 ml) for growth analyses as quickly as possible (15–30 min), refilled with media to get completely filled serum bottles and again compressed within a few minutes. Pressurized samples were incubated between 1 and 16 days depending on growth behavior. Growth at hydrostatic pressure was defined to be positive after two independent successful experiments. Growth at 0.1 MPa was assessed by using the same protocol except for pressurization. As the assays at 0.1 MPa were treated in the same way, an effect of dilution can be neglected. In general, all assays were carried out in triplicate and repeated at least twice.

### Hydrostatic Pressure Effects on Growth of *D. indonesiensis* Strain P23

To record the growth behavior of *D. indonesiensis* strain P23, pressure incubations were performed as described above. Growth curves and specific growth rates in response to different hydrostatic pressures were assessed by comparing the amount of sulfide and protein formed during defined times of incubation at low and high temperature. Specific growth rates were calculated from three to five data points along the logarithmic slope of the exponential portion of sulfide and protein curves using linear regression analysis.

The upper temperature limit for growth of *D. indonesiensis* strain P23 under high pressure was determined as follows: In pre-experiments, growth was tested in the range of 45 to 62°C at 20, 26, and 30 MPa. To reach the upper limit, growth curves were finally recorded in parallels at both, 0.1 and 20 MPa with slowly increasing temperatures from 45 to 52°C. Cultures grown at 45°C were allowed to adapt to higher temperatures for nine hours before incubation at 48°C. Temperature was increased again to 50°C after 12 h and to 52°C after 36 h of incubation. Pressure vessels were decompressed for growth analyses at the end of each temperature step as described above. After subsampling, serum bottles were refilled with fresh medium to circumvent substrate limitation.

### Cultivation and Extraction for Lipid Analysis

For determination of whole cellular FAs and intact polar lipids (IPLs) *D. indonesiensis* strain P23 was grown as described above at 20, 35, and 45°C at both, 0.1 and 30 MPa, in total culture volumes of 1.5 to 2.2 l. To obtain enough cell material, all pressure incubations were performed in parallels of up to 30 serum bottles using several pressure cylinders. To compensate for growth phase differences (Hamamoto et al., 1994; Allen et al., 1999), cells of each experiment were immediately harvested at late exponential growth phase, combined by centrifugation at 4°C, and stored at -20°C until further analyses. Total lipids were obtained by ultrasonic extraction from each washed cell pellet following a modified Bligh & Dyer procedure (Sturt et al., 2004) as described by Logemann et al. (2011). The lipid extracts were combined and evaporated to dryness under nitrogen at room temperature, stored at -20°C and analyzed by combined gas chromatography and mass spectrometry (GC–MS).

### Cellular FAs

The technical procedures were adapted from Rütters et al. (2002). In detail, aliquots of the total lipid extracts were transesterified with trimethylsulfonium hydroxide as described by Müller et al. (1990). FA methyl esters obtained were quantified by using a gas chromatograph (7890A GC-System Agilent Technologies, Santa Clara, CA, USA) equipped with a flame ionization detector (FID) and a capillary column (DB-5HT, length 30 m, ID 0.25 mm, 0.1 μm film thickness; J&W Scientific, Folsom, CA, USA). Identification was performed on a GC-MS system consisting of an HP 5890 Series II gas chromatograph (Hewlett Packard, Waldbronn, Germany) equipped with a DB-5HT column and coupled to a Finnigan MAT SSQ 710B mass spectrometer (Finnigan-Thermoquest, San Jose, CA, USA). Helium with a constant pressure of 12 psi was used for both systems. The GC oven temperature was raised from 60°C (isothermal for 2 min) to 360°C at a rate of 3°C min^-1^ and held for 5 min. Mass spectra were collected in full scan mode (*m/z* 50–650, ionization energy 70 eV, and 230°C source temperature). Mass spectrometric investigations were used to confirm the results obtained with GC-FID. FAs were identified by comparison of the retention times with those of known standards (Bacterial Acid Methyl Esters CP Mix; Supelco, Bellefonte, PA, USA).

### Intact Polar Lipids

Intact polar lipids were analyzed from an aliquot of each cell extract using high-performance liquid chromatography (HPLC)–electrospray ionization (ESI)-MS in the negative ion mode as described by Logemann et al. (2011). MS/MS spectra and full scan mass spectra (*m/z* 100–2000) were used for identification of head groups, diacylglycerols (DAGs) or acyl/ether glycerol (AEG) core lipids as well as fatty acyl side chains. Quantification was achieved by using an external multipoint calibration via compound mass trace areas. Phosphatidic acid (PA), phosphatidylglycerol (PG), phosphatidylethanolamine (PE), and phosphatidylinositol (PI) [all from Avanti Polar Lipids, Alabaster, AL, USA; Matreya, Pleasant Gap, PA, USA; Sigma–Aldrich, München, Germany, or Lipid Products, Redhill, UK] were used as standard compounds representative for different IPL classes. Due to the lack of commercially available standards for ornithine lipids (OL) or unknown polar lipids, OL were quantified via the calibration curve for PG (Seidel et al., 2013), while for unknown lipids the average signal response of all standards at every concentration was used.

## Results

### Growth of Sulfate-reducing Strains under High Hydrostatic Pressure

All Gram-negative sulfate-reducing pure cultures we had previously isolated at atmospheric pressure from subseafloor sediments (Fichtel et al., 2012) exhibited growth under elevated hydrostatic pressure of up to 30 MPa and 35°C (Supplementary Table S1). This was also found for the type strain of *D. indonesiensis* (Ind1^T^), which had originally been isolated from a corroding ship at the sea surface (Feio et al., 1998). In contrast, for *Desulfosporosinus lacus* strain P26, spore formation was induced by elevated pressure of up to 20 MPa. Only spores or sporulating cells were observed 10 days after incubation of freshly grown cells. This finding indicates that the original isolate may have been derived from a spore that germinated during the isolation procedure.

**FIGURE 1:**
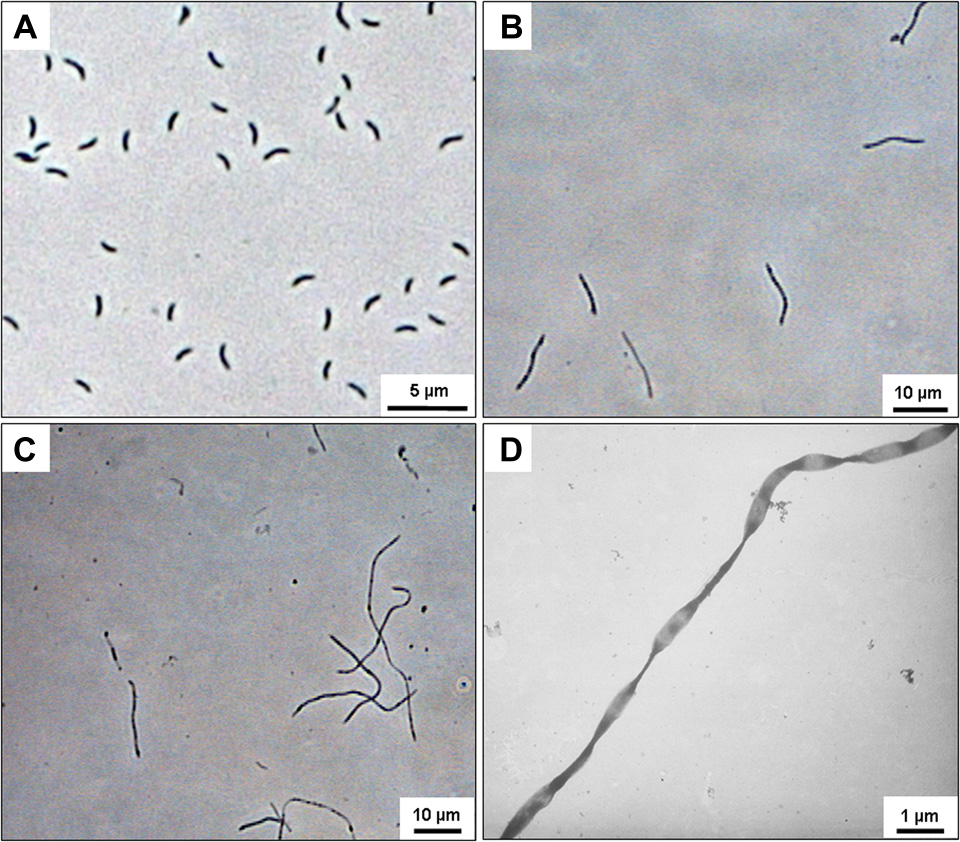
**Microscopic images of *Desulfovibrio indonesiensis* strains P12 and P23 grown at 45°C.** Phase-contrast image of *D. indonesiensis* strain P23 grown at 0.1 MPa **(A)** and at 40 MPa **(B)**. Phase-contrast image **(C)** and transmission electron microscopy (TEM) **(D)** of *D. indonesiensis* strain P12 grown at 40 MPa.

The combination of the highest pressure and temperature applied (40 MPa/45°C) severely affected the shape of *D. indonesiensis*-like strains P12 and P23. Instead of forming the typical motile, vibrio-shaped cells (**Figure 1A**) (Fichtel et al., 2012), cell division appeared to be incomplete, and both strains grew as long, straight, or twisted filaments (**Figures 1B–D**). Also, no cell motility was observed during microscopic inspection of the long filaments. Cultures of *D. indonesiensis* strain P23 grown at 45°C revealed a cell length from 1–1.7 μm (±0.17 μm) at atmospheric pressure which increased to an average of 14.7 μm (±5.18 μm) at 40 MPa (*n* = 20). Consequently, growth in further experiments was determined via sulfide formation and by measuring protein production rather than cell counting.

### Specific Growth Rates of *D. indonesiensis* Strain P23

**FIGURE 2:**
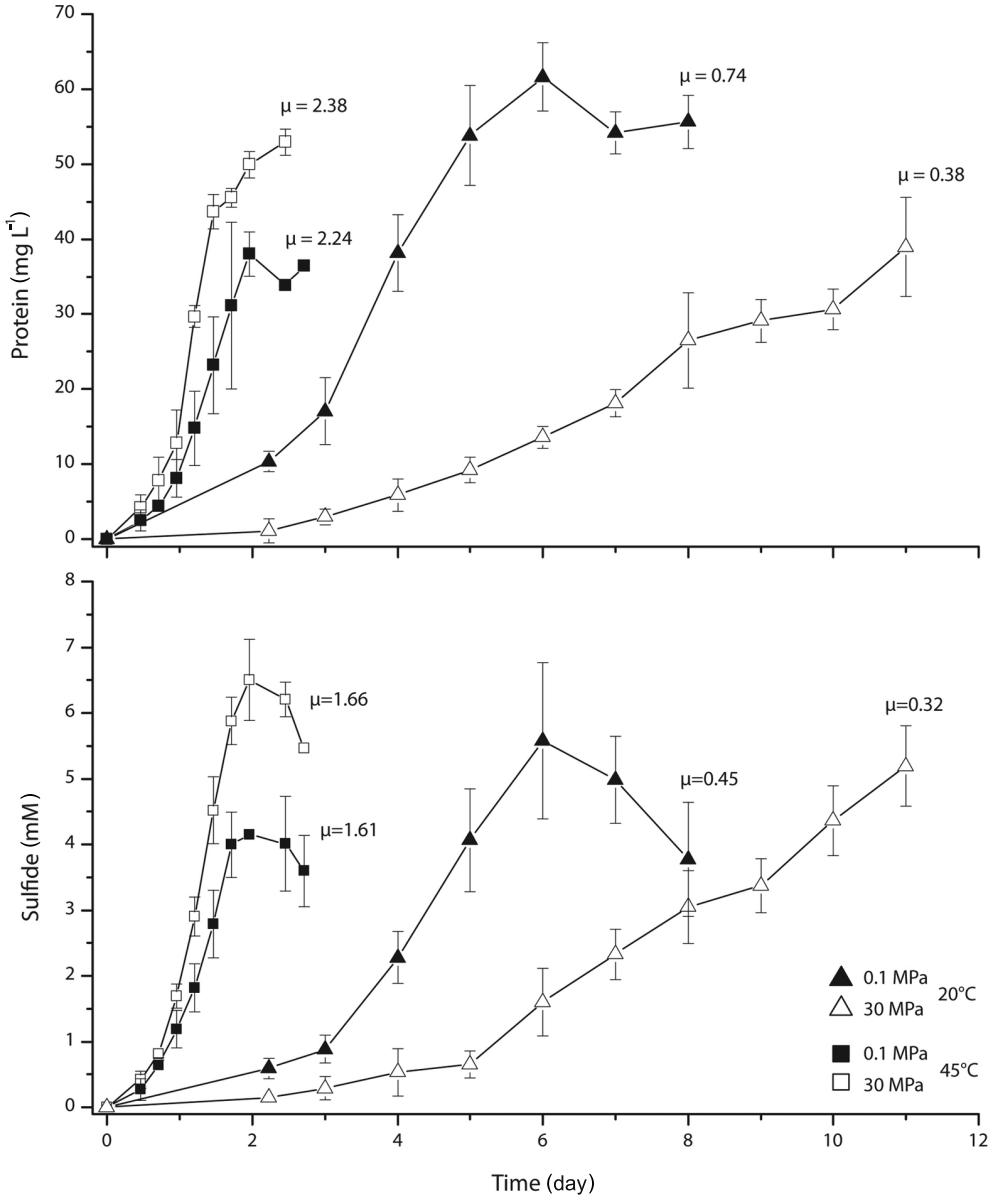
**Growth curves and specific growth rates μ (day^-1^) of *D. indonesiensis* strain P23 grown at atmospheric and high hydrostatic pressure both at 20 and at 45°C.** Values were calculated from photometrical measurements of protein and sulfide. Error bars indicate the standard deviation of three cultivation assays.

During cultivation experiments with *D. indonesiensis* strain P23 at various pressures (0.1, 10, 20, 30, and 40 MPa) and low or high temperatures (20 or 45°C), we found that increasing pressure reduced the specific growth rate at 20°C. At 45°C and pressures between 10 and 30 MPa, growth appeared to be faster as higher protein contents were determined after a given incubation time (data not shown). Growth curves were recorded in detail for cultures grown to early stationary phase at 20 and 45°C both, at atmospheric conditions and 30 MPa (**Figure 2**). Based on protein production, growth at 20°C was twice as fast at 0.1 MPa as under high-pressure conditions. The exponential growth rate μ was calculated as 0.74 day^-1^ at atmospheric pressure and 0.38 day^-1^ at high pressure. In contrast, growth rates obtained at 45°C under high-pressure conditions (2.38 day^-1^) were very similar to those at atmospheric pressure (2.24 day^-1^). A similar trend was determined via sulfide measurement. Comparing all rates, fastest growth was found at 45°C and 30 MPa, indicating the stimulation of growth by both, high temperature and *in situ* pressure. Highest protein yields were obtained at combinations of low temperature and atmospheric pressure (62 mg l^-1^) as well as high temperature and *in situ* pressure (53 mg l^-1^). Interestingly, the opposite combination of low temperature and high pressure with ~40 mg l^-1^ revealed a similarly low yield as at high temperature and low pressure.

### Pressure-induced Shift of the Maximum Growth Temperature

During a cultivation experiment to determine the upper temperature limit of growth at elevated hydrostatic pressure (20 MPa), cells were allowed to adapt to slowly increasing temperatures (Supplementary Figure S1). After 3 days of incubation in concert with a temperature shift to 50°C, cell counts in pressurized assays were highest at 50°C, reaching 4.04 × 10^8^ cells ml^-1^. In contrast, cells grown at 0.1 MPa reached their maximum at 48°C (2.23 × 10^8^ cells ml^-1^). Under these conditions, the previously determined maximum growth temperature of 48°C at atmospheric pressure was shifted to 50°C. At temperatures higher than 50°C, neither an ongoing protein production nor increasing sulfide formation was observed. This was confirmed in further test series that were inoculated with cells freshly grown at 50°C and 20 MPa. The upper temperature limit for growth was again indicated by the observation of highly elongated, non-motile, or deformed cells in comparison to the respective assays at 0.1 MPa.

### Changes in Whole-cell FA Composition as a Response to Increasing Temperature and Elevated Pressure

The majority of whole cell FAs of *D. indonesiensis* strain P23 were branched and accounted for up to 79% under atmospheric pressure (Supplementary Table S2). Regardless of growth temperatures and pressures, major components were *iso*- and *anteiso*-branched 15:0 and *n*-18:0 FAs, which was already known for the type strain (Feio et al., 1998). Concerning the degree of unsaturation, only monounsaturated FAs were detected.

**FIGURE 3:**
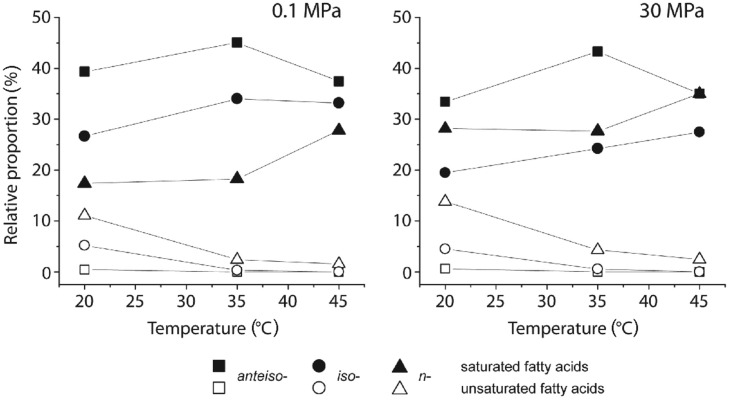
**Changes in relative amounts of whole cell fatty acids (FAs) of *D. indonesiensis* strain P23 grown at different temperatures and pressures**.

In comparison to cells grown at atmospheric conditions and 20°C, *D. indonesiensis* strain P23 showed strongly elevated levels of *n*-saturated FAs at the expense of branched-saturated FAs during incubation at the same temperature but high pressure (**Figure 3**; Supplementary Table S2). Under both pressure regimes, a two-step response of *D. indonesiensis* strain P23 was detected for increasing incubation temperatures. First, the relative amount of unsaturated FAs decreased strongly. Second, levels of *n*-saturated FAs increased mainly at the expense of *ai*-branched-saturated FAs. Comparing temperature-dependent incubations under atmospheric and under *in situ* pressure, pressure did not substantially increase the ratio of unsaturated to saturated FAs, but led to a higher *n*-saturation and concomitant decreased branching of FAs. Additionally, relative proportions of longer-chain FAs were substantially elevated under high-pressure conditions only (Supplementary Table S2).

### Relative Distribution of Main IPLs Depending on Temperature and Pressure

Under all conditions tested, *D. indonesiensis* strain P23 contained two classes of IPLs: phospholipids (PG, PE, and PA) and the phosphorus- and glycerol-free OL (Supplementary Figure S2; Supplementary Table S3a). Phospholipids mainly contained a DAG-core lipid with ester-bound FA moieties as identified by MS–MS experiments. Additionally, PG was also detected as AEG with mixed ether/ester-bound side chains. Four additional IPLs with unidentified head groups were found (Un1-4). It appears likely that they represent yet unknown phospholipids as they were also detected as DAG or AEG (Supplementary Figures S3 and S4; Supplementary Table S3b).

**FIGURE 4:**
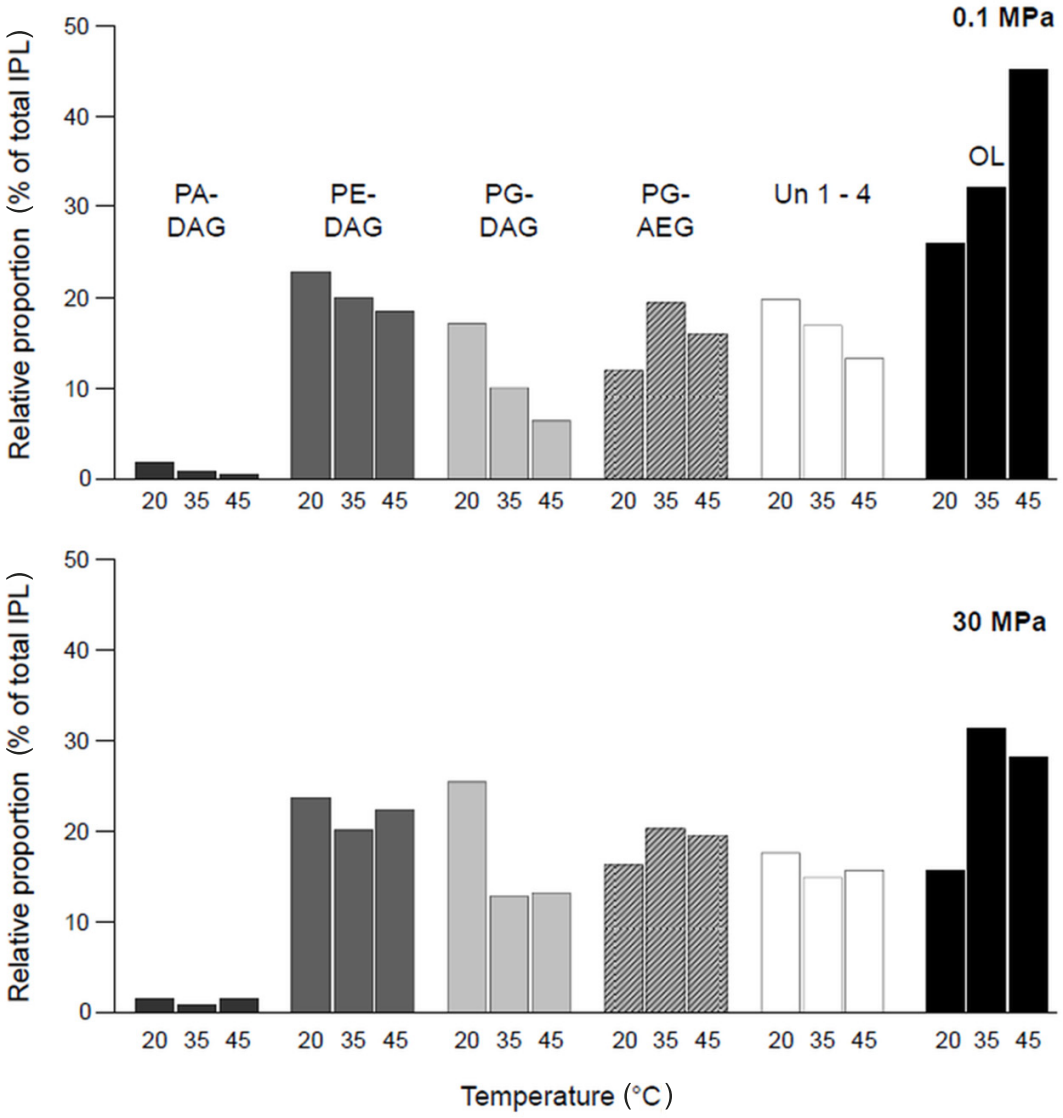
**Changes in relative amounts of major intact polar lipid (IPL) species of *D. indonesiensis* strain P23 depending on growth temperature and pressure**.

Comparing the IPL compositions in almost all assays (exception 0.1 MPa and 45°C), the relative proportion of all phospholipids dominated over OLs. At high pressure, this behavior was more pronounced (Supplementary Table S4). For all other experiments at atmospheric pressure with increasing incubation temperatures, the amount of OL increased, while relative proportions of all other IPL except PG-AEG decreased (**Figure 4**). At pressures of 30 MPa, no clear trend in relative IPL proportions was observed. Major shifts with increasing temperatures were found for diacyl-phosphatidylglycerol (PG-DAG) and acyl-ether-phosphatidylglycerol (PG-AEG). While levels of PG-DAG dominated over PG-AEG at low temperature, the opposite ratio was found at higher temperatures. The values of total unknown IPLs (Σ Un 1–4) showed a response to temperature changes similar to that of the phospholipids. In high-pressure cultures, the effect of increasing temperature on the IPL composition was most pronounced between 20 and 35°C.

### Composition and Length of IPL Side Chains Change with Cultivation Pressure and Temperature

The majority of analyzed IPLs contained a FA with 15 carbon atoms (Supplementary Table S3a). Moreover, the C_15_-FA was the only FA component in IPLs with an AEG core. Polyunsaturated FAs were never detected. Ratios of unsaturated to saturated IPLs decreased with rising incubation temperature and were slightly higher in high-pressure assays. These findings are in good accordance with the whole cell FA data (Supplementary Table S2).

Side-chain combinations of PE and PG were quite similar with either C_14_- or C_15_-FA together with a C_15_–C_20_-moiety at the *sn*-2 position. Interestingly, two unknown IPLs (Un-1 and Un-2) contained either a fairly long FA chain or an ether-bound alkyl moiety of 21–23 carbon atoms. OLs had either 14:0 or 15:0 FAs together with a 3-hydroxy C_16_–C_20_-FAs.

**FIGURE 5:**
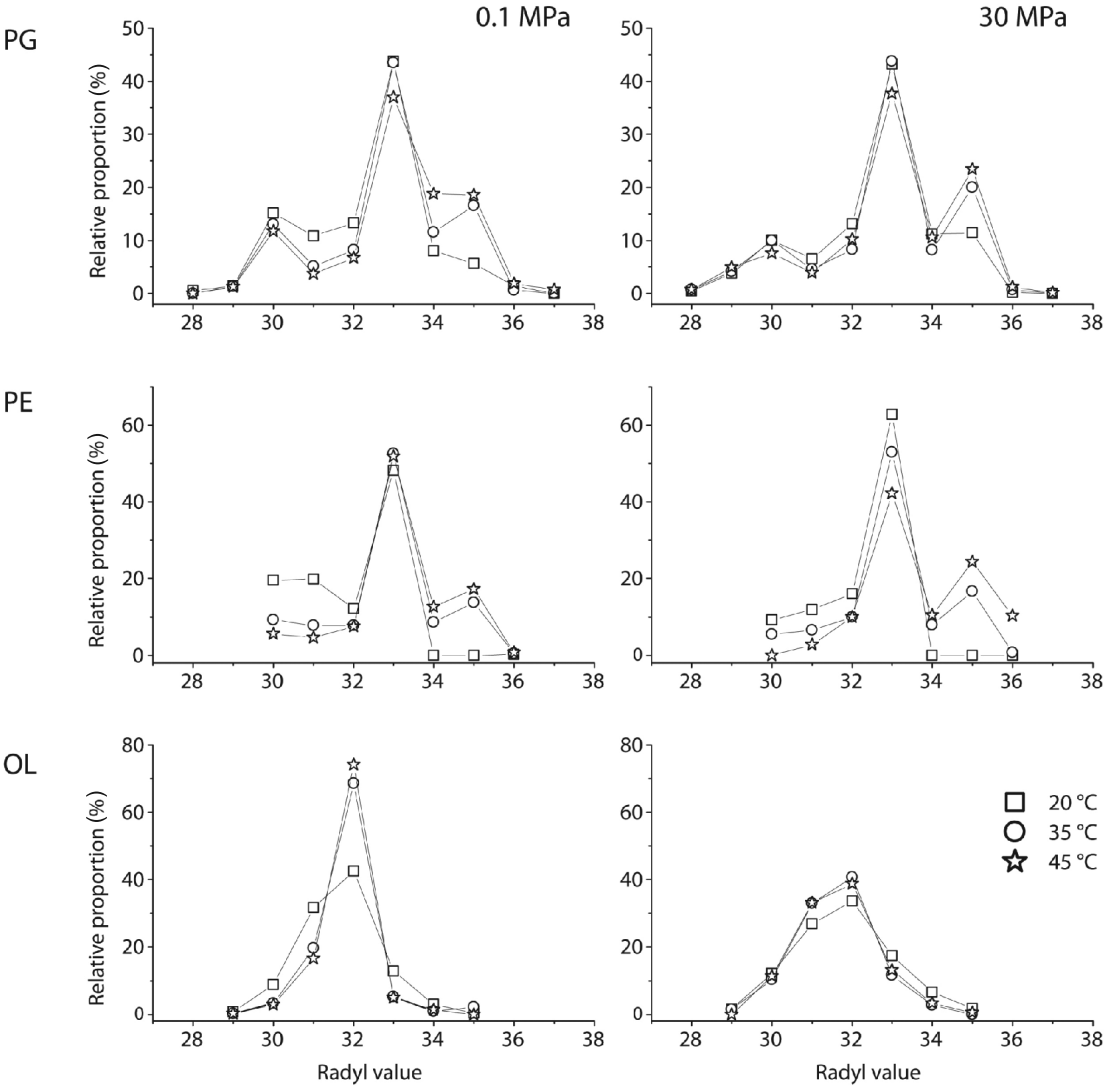
**IPLs inventory of strain P23 depending on different incubation conditions**. Changes in fatty acyl side-chain length are expressed by radyl values, the combined number of carbon atoms of the two FA substituents of one IPL type. PG, phosphatidylglycerol; PE, phosphatidylethanolamine, and OL, ornithine lipid. PG data is based on the sum of PG-DAG and -AEG.

Cell response of *D. indonesiensis* strain P23 to pressure and temperature was either reflected in changes of abundance, saturation, or carbon-number distribution of fatty acyl side chains in the three major polar lipids PE, PG, and OL. However, focusing on a single mass of an IPL without MS/MS experiments, several possibilities for the combination of ester- or ether-linked moieties arise. To simplify our data, we used the radyl value comprising the total carbon number of both side chains. As a result, PG generally had the greatest diversity of side chains, which was reflected in the broad range of radyl values from 28 to 37. Radyl values in PE varied between 30 and 36, and between 29 and 35 in OL. The radyl value pattern for PE and PG was dominated by 33 carbon atoms, resulting from high proportions of C_15_- and C_18_-FAs, while that of OL was dominated by 32, resulting from C_15_- and 3-OH-C_17_-FAs (**Figure 5**).

For PE and PG, higher incubation temperatures induced an increase of longer side chains at the expense of shorter ones. Additionally, at high-pressure incubations, the most abundant PE molecular species with a radyl value of 33 systematically decreased with temperature. Most strikingly, the radyl value pattern of OL was not affected by temperature during high-pressure incubations. Here, the distribution patterns were nearly identical and similar to that obtained at 20°C and 0.1 MPa. In contrast, major structural changes were found at atmospheric pressure and high temperatures with a dramatic increase of the relative proportion of the most dominant OL species with a radyl value of 32 carbon atoms.

In general, levels of unsaturated side chains in all major IPLs were highest at 20°C and decreased strongly with rising incubation temperature. While PE-DAG lipids contained the largest proportion of unsaturated moieties, PG lipids had the greatest diversity of side chains. As the PG pool even showed a temperature-induced restructuring with an internal shift from PG-DAG to PG-AEG, findings indicate that in *D. indonesiensis* strain P23 restructuring of PE and PG was relevant for bilayer stabilization, as a result of both, temperature and pressure changes.

## Discussion

Reflecting the *in situ* conditions, our isolate P23 grew fastest at combined high pressure and high temperature. Remarkably, the cells responded to a temperature rise in a much more pronounced way than to elevated pressure. By decreasing pressure and temperature simultaneously, it was even possible to cultivate our moderately piezothermophilic strain from the warm deep subsurface at ambient conditions.

### High-pressure Experiments Reveal the Piezothermophilic Nature of *D. indonesiensis* Strain P23

A high-pressure tolerance should demonstrate that micro organisms of the deep biosphere are well adapted to their pressurized subsurface habitat and that they do belong to the active part of deeply buried microbial communities (Bale et al., 1997). In this study, we demonstrated that *D. indonesiensis* strain P23 was able to grow under hydrostatic pressure of up to 40 MPa (*in situ* pressure ~30 MPa), even after cultivation at atmospheric pressure for more than 3 years. *D. indonesiensis* strain P23 obviously has not lost its piezophilic properties, which may be a common feature of pressurized deep-sea organisms sampled and isolated under decompressed conditions (Zobell and Johnson, 1949).

The degree of piezophily was strongly dependent on the incubation temperature (Zobell and Johnson, 1949; Kato et al., 1995). In our previous study (Fichtel et al., 2012), *D. indonesiensis* strain P23 was found to have an upper growth temperature of 48°C. At 20°C, *D. indonesiensis* strain P23 grew at both, atmospheric and elevated hydrostatic pressure with fastest growth rates at 0.1 MPa. In contrast, at higher temperatures, growth was accelerated by high hydrostatic pressure in the broad range from 10 to 30 MPa, and the maximum temperature of growth rose to 50°C only under pressure. Under these conditions, strain P23 would be considered to be moderately piezothermophilic (Yayanos, 1995; Kato and Bartlett, 1997) reflecting its adaptation to the *in situ* conditions present in its original warm subsurface habitat.

However, an increase of growth temperature does not necessarily improve the piezotolerance of microorganisms. For instance, in a study on typical atmosphere-adapted lactic acid bacteria, higher temperatures did not stimulate microbial growth under elevated pressure (Molina-Höppner et al., 2003). Unlike many pressure-adapted species, these lactic acid bacteria and other mesophiles may be unable to develop a specific pressure response in order to maintain membrane fluidity.

During growth at conditions above *in situ* pressure, the cell shape of our isolates changed. With increasing pressure and temperature, cells became more elongated and cell division was inhibited, indicating a typical stress response. Cell filamentation appears to be a characteristic pressure-related phenomenon in mesophilic bacteria (Zobell and Cobet, 1962, 1964; Lauro and Bartlett, 2008). Pressure is believed to have a direct inhibitory effect on FtsZ ring formation, which is a prerequisite for membrane construction during cell division (Molina-Höppner et al., 2003; Ishii et al., 2004). Filament formation may also be mediated via a pressure-triggered SOS response involving the RecD protein, which is essential for DNA recombination and repair (Bidle and Bartlett, 1999; Aertsen et al., 2004). Interestingly, filamentation has frequently been shown to occur in piezophilic bacteria (e.g., *Marinitoga piezophila*) grown at atmospheric pressure (Alain et al., 2002). Our inverse finding may indicate that filamentation is a more general stress response that is triggered by pressure but works in both directions.

### Pressure and Temperature Effects on Membrane Lipid Composition

While the whole cell FA analysis provides a fast overview on all cellular FAs, the analysis of IPLs directly targets the membrane building blocks. The results of both methods can be combined as FAs extracted from the membrane fraction are very similar to those extracted from whole cells (Pluschke and Overath, 1981; Allen et al., 1999; Kaneko et al., 2000).

Due to the theory of ‘homeoviscous adaptation of membrane lipids’ (Sinensky, 1974; Somero, 1992), it was expected that increasing growth temperature mainly results in a higher degree of saturation of membrane lipids to keep them appropriately fluid for integrity and cell function. For *D. indonesiensis* strain P23, this adaptation was detected with both analytical methods confirming previous studies on a variety of organisms (DeLong and Yayanos, 1985). Moreover, the cell response of *D. indonesiensis* strain P23 to changes in temperature apparently occurred stepwise. After changing the saturation level, as a subsequent response to higher incubation temperature *D. indonesiensis* strain P23 decreased its membrane fluidity by exchanging branched FAs with straight-chain FAs. This was most pronounced for *anteiso*-branched FAs, as they have lower melting points than *iso*-branched FAs; the effect is similar to that of saturation (Zhang and Rock, 2008). For *D. indonesiensis* strain P23, elevated initial proportions of straight-chain FAs under *in situ* pressure and 20°C were independent of the degree of saturation and indicated that pressure mostly diminished the branching of FAs. These findings are consistent with results obtained in previous studies on thermal adaptation of bacterial membranes (Rilfors et al., 1978; Nordström and Laakso, 1992; Koga, 2012). Comparing low and high-pressure incubations at the same temperatures, *D. indonesiensis* strain P23 reacted similarly as described for many other microorganisms with decreasing proportions of saturated FAs at high pressure (Fang et al., 2003). An initially higher ratio of unsaturated over saturated FAs (Wang et al., 2014) was not observed.

Another factor for membrane fluidity is the number of double bonds of unsaturated FAs. Polyunsaturated FAs were found in many piezopsychrophilic deep-sea bacteria, this regulatory capacity appears to be limited to psychrophilic microorganisms (DeLong and Yayanos, 1986; Wirsen et al., 1986; Kamimura et al., 1993; Yano et al., 1998) or mesophilic organisms from near-surface sediments (Freese et al., 2009). As *D. indonesiensis* strain P23 derives from a warm deep-sea habitat, the production of polyunsaturated FAs as a regulatory component may only become relevant when growth temperature falls below 20°C.

### High Proportions of Ornithine-containing Lipids may Reflect Phosphate Limitation in the Original Habitat

Phosphorus-free ornithine-containing lipids are major membrane constituents in *D. indonesiensis* strain P23, as already described for other *Desulfovibrio* species (Makula and Finnerty, 1975; Seidel et al., 2013). The presence of OLs was found to be negatively correlated with available amounts of phosphate present in the culture medium (Geiger et al., 1999; Weissenmayer et al., 2002). Thus, the authors assumed that bacteria replaced phosphate-containing membrane lipids by phosphorus-free lipids such as OL, sulphoquinovosyl diacylglycerol (SQDG), or diacylglycerol trimethylhomoserine (DGTS). Although phosphate was not a limiting nutrient in our cultivation medium, *D. indonesiensis* strain P23 was isolated from phosphate-depleted sediment layers (Engelen et al., 2008). In this environment, the ability to produce phosphate-free membrane building blocks gives an advantage over other microorganisms that are not capable of this regulatory feature. Thus, it is possible, that this adaptation originally developed in the deep biosphere and was not lost when *D. indonesiensis* strain P23 was cultivated in phosphate-rich media.

Furthermore, our study indicates that OL may not only be a substitute for phosphate-containing membrane lipids. Cells grown at atmospheric pressure responded to increasing temperature with higher relative amounts of OL within the membrane, and FA chain-length variations. This temperature dependence corroborates recent findings by Seidel et al. (2013) who found elevated proportions of OL with increasing incubation temperature for several *D. acrylicus* strains. The authors suggested that changes in lipid composition of the cytoplasmic membrane may rather be important for the presence and activity of membrane-bound enzymes and do not necessarily influence viscosity. This assumption is supported by our results, as we found neither the expected decrease of OL levels with temperature in high-pressure incubations nor any obvious structural changes of OL moieties as an adaptation of membrane fluidity to high pressure.

### Simultaneous Decrease of Pressure and Temperature Favors the Cultivation of Piezomesophiles from Deep Subsurface Sediments

To mimic environmental conditions, most enrichment cultures are incubated at *in situ* temperatures. In contrast, even for the cultivation of deep-sea microorganisms, pressure is normally not taken into account. This may be due to the high technical effort during sampling, storage, and microbial analyses (Parkes et al., 2009). While none of our enrichments from IODP Site U1301 that were performed at *in situ* temperatures and atmospheric pressure resulted in pure cultures, a great variety of isolates were gained at ambient laboratory conditions (Fichtel et al., 2012). This can be explained by opposed effects of pressure and of temperature on general cell functions and the capability of microorganisms of adapting to these variations. Thus, a key for a successful isolation of piezomesophilic and other high-temperature-adapted prokaryotes from the subsurface under atmospheric pressure appears to be the decrease of cultivation temperature below the *in situ* temperature. For piezopsychrophiles in turn, this approach is not appropriate as *in situ* conditions of, e.g., 40 MPa and 2°C, the average values at the seafloor, would require an unrealistic incubation temperature of -2 to -6°C at atmospheric pressure (Chong and Cossins, 1983; Bartlett, 2002; Wang et al., 2009). On the other hand, pressure above *in situ* values may be advantageous for cultivating piezopsychrophiles at elevated temperatures, which generally accelerates growth (Kato et al., 1995).

In case of *D. indonesiensis* strain P23, applying the optimum hydrostatic pressure resulted in growth at 50°C but not at the respective *in situ* temperature of ~60°C. However, this *in situ* temperature may not be precise. Temperatures at depth were estimated only by extrapolating heat flow data. They are estimated based on the assumption that heat transport within the sediment is vertical and conductive. This leads to uncertainties in temperatures estimated for the sediment–basement interface of ~15–25% (Zühlsdorff et al., 2005). Another explanation why the *in situ* temperature was not reached is possibly due to the fact that the chemical composition of our media did not reproduce the chemical condition of the natural habitat. Alternatively, it may be explained by the effect of pressure on abiotic factors such as the solubility of gases. *D. indonesiensis* strain P23 turned out to be a chemolithoautotrophic sulfate reducer as it was able to grow on hydrogen and CO_2_ as sole energy and carbon sources (Fichtel et al., 2012). In the present study, pressure incubation under autotrophic conditions was not performed, as this would have required a completely different technical equipment allowing cultivation with a gaseous headspace. However, pressure is an inevitable factor for isolating obligate piezophiles (Kato et al., 1996) or investigating the microbial utilization of gases or hydrocarbons in the subsurface.

## Author Contributions

KF: Idea and concept, high-pressure experiments, data analyses (calculations, creation of tables and figures), microscopic pictures, data interpretation, first draft; JL: Idea and concept, analyses of whole-cell fatty acids and intact polar lipids, data analyses (calculations, creation of tables and figures), data interpretation, first draft; JF: Lipid analyses, data interpretation, revision; BE: Sampling, idea and concept, data interpretation, revision; HC: Idea and concept, data interpretation, revision; JR: Data interpretation, revision.

## Conflict of Interest Statement

The authors declare that the research was conducted in the absence of any commercial or financial relationships that could be construed as a potential conflict of interest.
