# Supplementary material for: Temperature and pressure adaptation of a sulfate reducer from the deep subsurface
Source: Front Microbiol. 2015 Oct 6;6:1078. doi: 10.3389/fmicb.2015.01078 (PMC4594026; doi:10.3389/fmicb.2015.01078)
Supplement: Supplementary file 1 [file Data_Sheet_1.PDF]

Supplementary information to:

Temperature and pressure adaptation of a sulfate reducer from the deep subsurface

Katja Fichtel[1], Jörn Logemann[2], Jörg Fichtel[2], Jürgen Rullkötter[2], Heribert Cypionka[1] and Bert Engelen[1*]

[1] Paleomicrobiology Group, Institute for Chemistry and Biology of the Marine Environment, University of Oldenburg, Oldenburg, Germany
[2] Organic Geochemistry Group, Institute for Chemistry and Biology of the Marine Environment, University of Oldenburg, Oldenburg, Germany

Running title:
Piezophilic sulfate-reducing subsurface bacteria

[*]Correspondence:
Dr. Bert Engelen
Institute for Chemistry and Biology of the Marine Environment
Paleomicrobiology
Carl-von-Ossietzky-Straße 9-11
26129 Oldenburg, Germany
Email: engelen@icbm.de

Further remarks:
Authors K. Fichtel and J. Logemann contributed equally to this work.

Table S1: Summary of strains used for comparison in this study. Phylogenetic affiliation, isolation depth, *in situ* conditions, temperature range of growth and investigated maximum growth pressures and temperatures are given.

Strains used in this study	Closest relative in GenBank / sequence similarity	Depth (mbsf)	*In situ* T (°C)	*In situ* P* (MPa)	T_{range} of growth at 0.1 MPa (°C)	Growth at P?	P / T max. tested / days of incubation
P26	*Desulfosporosinus lacus*[T] / 97%	1.3	2.2	26.6	n. d.	no	20 MPa / 25°C / 10 d
P20	*Desulfovibrio aespoeensis*[T] / 98%	1.3	2.2	26.6	20-35	yes	30 MPa / 35°C / ~4 d
P34	*Desulfovibrio indonesiensis*[T] / 99%	239.5	56.4	29.0	10-48	yes	30 MPa / 45°C / ~2 d
P12	*Desulfovibrio indonesiensis*[T] / 99%	251.9	59.2	29.1	10-48	yes	40 MPa / 45°C / 1.5 d
P23	*Desulfovibrio indonesiensis*[T] / 99%	260.4	61.2	29.2	10-48	yes	40 MPa / 60°C / var.
P18	*Desulfotignum balticum*[T] / 99%	260.4	61.2	29.2	4-48	yes	30 MPa / 35°C / ~16 d
Type strain	*Desulfovibrio indonesiensis*[T], strain Ind 1	Surface	n.k.	0.1	10-37	yes	30 MPa / 35°C / ~2 d

n.d., not determined; n.k., not known; *based on water depth (2656 m) and depth of sediment sample

Table S2: Whole cell fatty acid composition of *D. indonesiensis*-affiliated strain P23 grown at three different temperatures at constant atmospheric and high hydrostatic pressure. Single values represent relative amounts in % of total fatty acids, while types with all parts <1 % are not listed. Fatty acids are commonly designated by number of carbon atoms:number of double bonds. *Iso*- and *anteiso*-branching refer to the prefixes "*i*" and "*ai*", respectively, "*n*" symbolizes straight and saturated chains. ∑ = Sum of all measured values.

**Fatty acid**		**0.1 MPa**			**30 MPa**	
	**20°C**	**35°C**	**45°C**	**20°C**	**35°C**	**45°C**
i-14:0	6.4	4.2	3.0	5.2	5.4	6.4
n-14:0	2.1	2.6	2.8	4.8	5.1	6.8
i-15:1	1.6	-	-	2.1	0.5	-
i-15:0	16.2	24.9	24.5	11.1	15.9	18.4
ai-15:0	39.3	45.1	37.0	33.5	43.3	35.0
i-16:0	2.0	1.0	0.7	1.6	1.1	1.0
n-16:0	4.2	4.1	6.7	7.2	4.3	5.9
i-17:1	1.6	-	-	0.9	-	-
i-18:0	1.3	1.1	1.0	1.0	0.8	0.6
n-18:1	8.2	1.0	0.9	7.1	1.9	0.8
n-18:0	10.3	8.5	14.7	14.3	11.9	11.7
i-19:1	2.1	0.3	-	1.5	-	-
i-19:0	0.3	1.7	3.0	0.2	0.6	0.5
n-20:1	2.9	1.1	-	6.8	2.4	1.3
n-20:0	0.5	2.9	3.2	1.3	5.9	9.2
Σ Unsaturated FAs	17	3	2	19	5	3
Σ Saturated FAs	83	97	98	81	95	98
Σ n-sat. FAs	17	18	28	28	28	35
Σ i-sat. FAs	27	34	33	19	24	28
Σ ai-sat. FAs	39	45	37	34	43	35
Σ Branched FAs	72	79	71	58	68	62
Unsat. / Sat.	0.20	0.03	0.02	0.23	0.05	0.03
Anteiso / iso	1.48	1.33	1.13	1.73	1.78	1.27

Table S3a: IPLs inventory of strain P23. Mass-to-charge ratio (*m/z*), linked fatty acid combinations and retention times of major molecular species identified by HPLC-ESI-MS-MS in negative ion mode. Abbreviations: OL, ornithine lipid; PE, phosphatidylethanolamine; PG, phosphatidylglycerol; PA, phosphatidic acid.

Lipid / Headgroup	*m/z*	Fatty acyl side chains	Retention time
	(negative ion mode)	(MS-MS analysis)	[min]
**OL**	609.5	15:0 / 3-OH 16:0<br>14:0 / 3-OH 17:0	8.8
	623.5	15:0 / 3-OH 17:0	8.6
	635.6	15:0 / 3-OH 18:1	9.3
	637.6	15:0 / 3-OH 18:0	9.3
	651.6	15:0 / 3-OH 19:0<br>14:0 / 3-OH 20:0	10.4
**PE**	676.5	15:0 / 15:0<br>14:0 / 16:0	11.3
	690.5	15:0 / 17:0<br>14:0 / 18:0	11.3
	702.5	15:0 / 18:1	11.3
	704.6	15:0 / 18:0	11.1
	718.6	15:0 / 19:0<br>14:0 / 20:0	11.0
	730.6	15:0 / 20:1	11.3
	732.6	15:0 / 20:0	11.4
**PG**	693.6	15:0 / 15:0<br>14:0 / 16:0	10.7
	719.5	15:0 / 17:1<br>14:0 / 18:1	10.2
	721.5	15:0 / 17:0<br>14:0 / 18:0<br>15:0 / O-18:0	9.5<br>9.5<br>7.8
	733.6	15:0 / 18:1<br>14:0 / 19:1	10.8
	747.5	15:0 / 19:1<br>14:0 / 20:1<br>16:0 / 18:1	10.7
	749.7	15:0 / 19:0<br>14:0 / 20:0<br>15:0 / O-20:0	10.3<br>10.3<br>9.7
	763.7	15:0 / 20:0	10.4
**PA**	661.6	15:0 / 18:0	
	675.6	15:0 / 19:0<br>14:0 / 20:0<br>16:0 / 18:0	7.1
	689.6	15:0 / 20:0<br>14:0 / 21:0	7.0

Table S3b: IPLs inventory of strain P23. Mass-to-charge ratio (*m/z*), linked fatty acid combinations and retention times of major molecular species identified by HPLC-ESI-MS-MS in negative ion mode. Abbreviations: Un1-4, four unidentified lipids, probably yet unknown phospholipid species.

**Lipid / Headgroup**	***m/z***	**Fatty acyl side chains**	**Retention time**
	(negative ion mode)	(MS-MS analysis)	[min]
**Un-1**	865.5	14:0 / 21:0	6.4
Mass of head group:	879.5	15:0 / O-23:0	4.8
162 Da	893.5	15:0 / 23:0	6.1
**Un-2**	867.4	15:0 / O-22:0	5.0
Mass of head group:	881.5	15:0 / 22:0	6.1
164 Da	895.5	15:0 / O-23:0	6.8
	909.7	15:0 / 23:0	6.9
**Un-3**	787.5	15:0 / 16:0	16.8
Mass of head group:	801.5		16.6
154 Da	813.6		16.8
	815.6		15.8
	829.6	15:0 / 19:0<br>14:0 / 20:0	15.6
	843.6	15:0 / 20:0	15.9
**Un-4**	787.5		15.7
Mass of head group:	801.5		14.7
168 Da	813.5	15:0 / 18:1	14.8
	815.6	15:0 / 18:0	14.7

Table S4: Major polar lipid types (in % of total IPLs, intact polar lipids) in strain P23 grown at three different temperatures at atmospheric and high hydrostatic pressure. Abbreviations: OL, ornithine lipid; PE, phosphatidylethanolamine; PG, phosphatidylglycerol; PA, phosphatidic acid; Core lipids: DAG, diacyl glycerol; AEG, Acyl-ether glycerol; Un1-4, four unidentified lipids, probably yet unknown phospholipid species.

**IPLs**	**0.1 MPa**			**30 MPa**		
	**20 °C**	**35 °C**	**45 °C**	**20 °C**	**35 °C**	**45 °C**
OL	26.1	32.2	45.2	15.7	31.3	28.1
Σ Phospholipids	54.1	50.7	41.5	66.8	53.9	56.5
PA-DAG	1.9	0.9	0.5	1.6	0.8	1.6
PE-DAG	22.9	20.2	18.5	23.7	20.1	22.3
PG-DAG	17.2	10.1	6.5	25.3	12.8	13.2
PG-AEG	12.1	19.5	16.0	16.2	20.2	19.4
Σ Un1-4	19.9	17.1	13.4	17.6	14.8	15.6

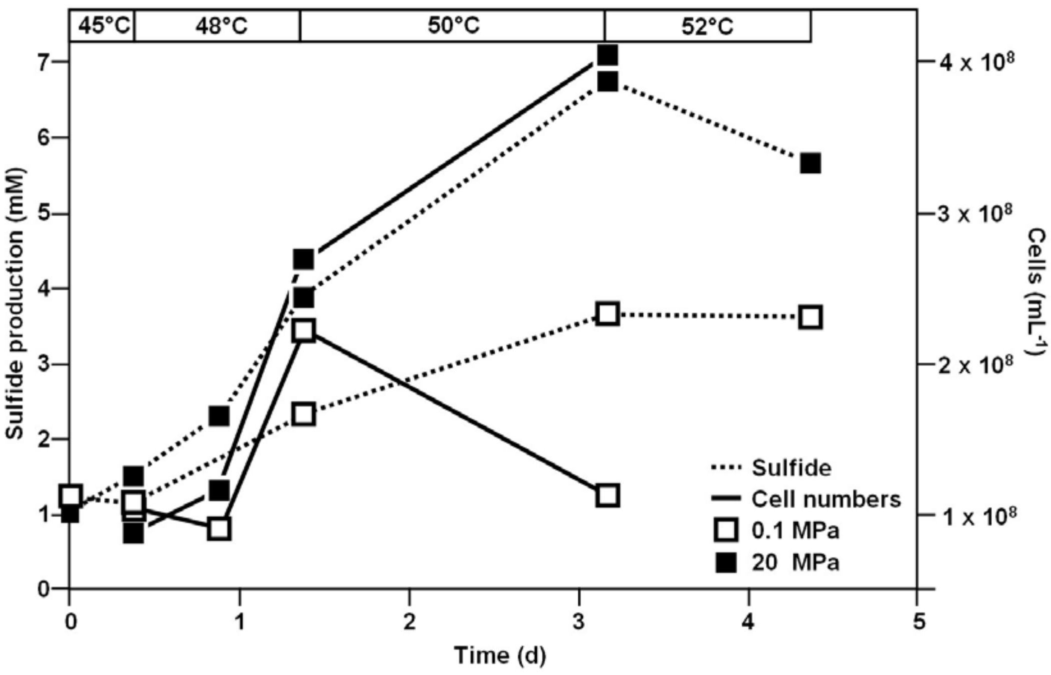

Figure S1: Cultivation experiment with *D. indonesiensis* strain P23 to determine the upper temperature limit of growth at 0.1 and 20 Mpa. Growth was assessed by cell counting and comparing the amount of sulfide formed. Cultures grown at 45 °C were allowed to adapt to higher temperatures for nine hours before incubation at 48 °C. Temperature was increased again to 50 °C after 12 h and to 52 °C after 36 hours of incubation.

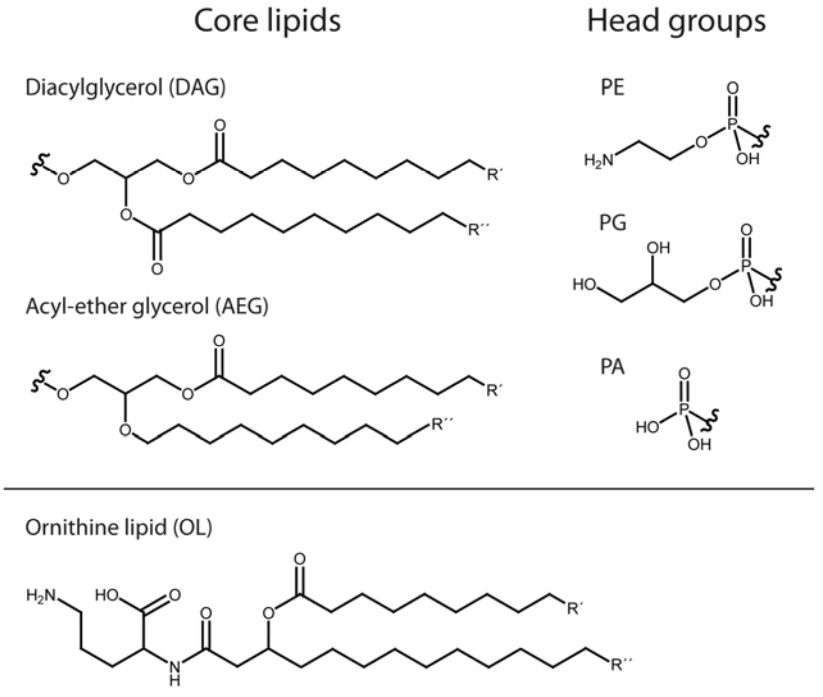

Figure S2: Molecular structure of major intact polar lipids present in *Desulfovibrio indonesiensis*-affiliated strain P23. OL, ornithine-containing lipid; Head groups of phospholipids: PE, phosphatidylethanolamine; PG, phosphatidylglycerol; PA, phosphatidic acid; Core lipids of phospholipids: Diacylglycerol, DAG with ester-bound fatty acid moieties; Acyl-ether glycerol, AEG with mixed ether/ester bound side chains. R', R'', alkyl moieties.

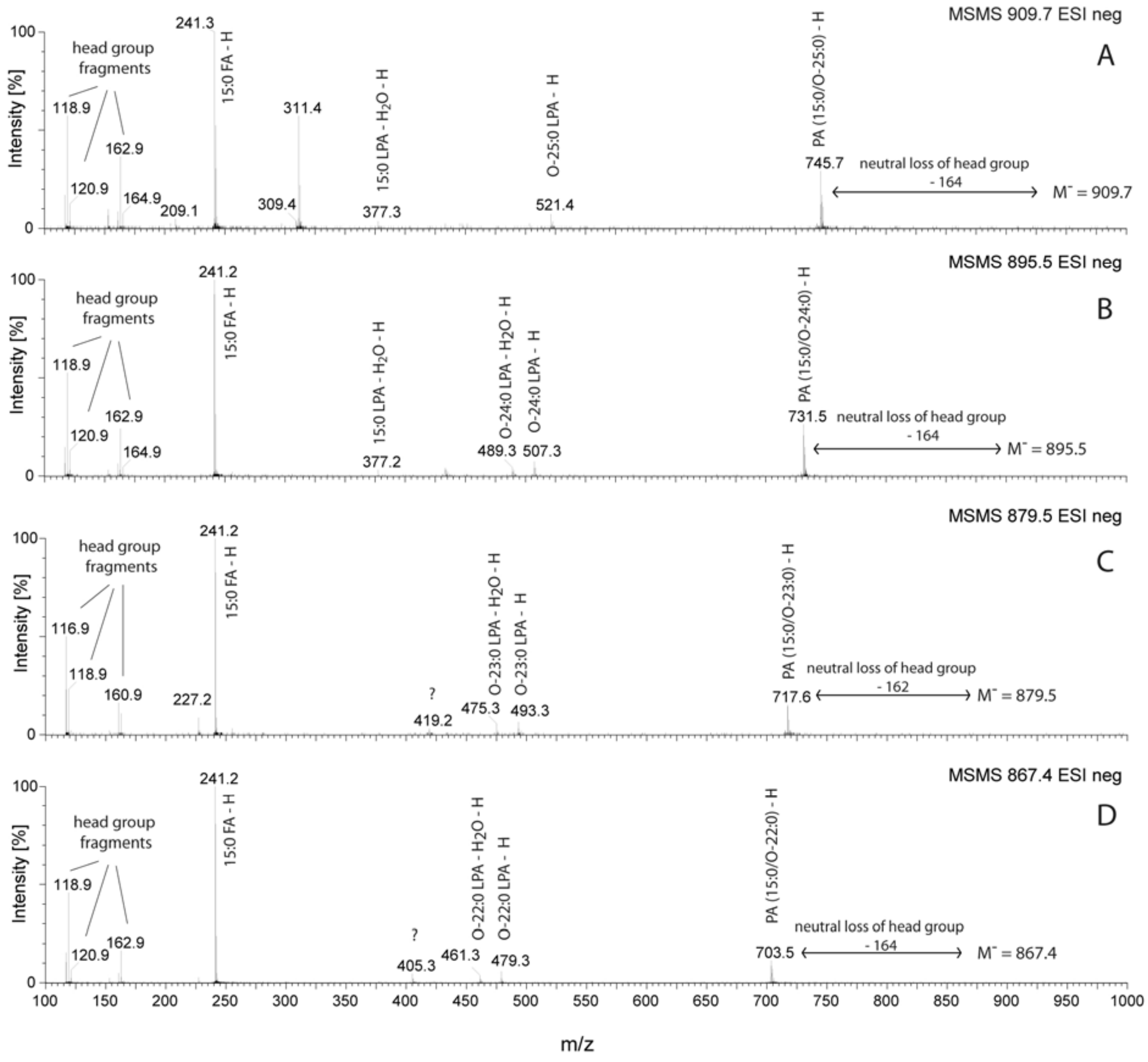

Figure S3: MS-MS-spectra of unidentified compounds Un-1 (C) and Un-2 (A, B, D) with two unknown head groups detected in strain P23. Spectra were recorded in ESI negative ion-mode.

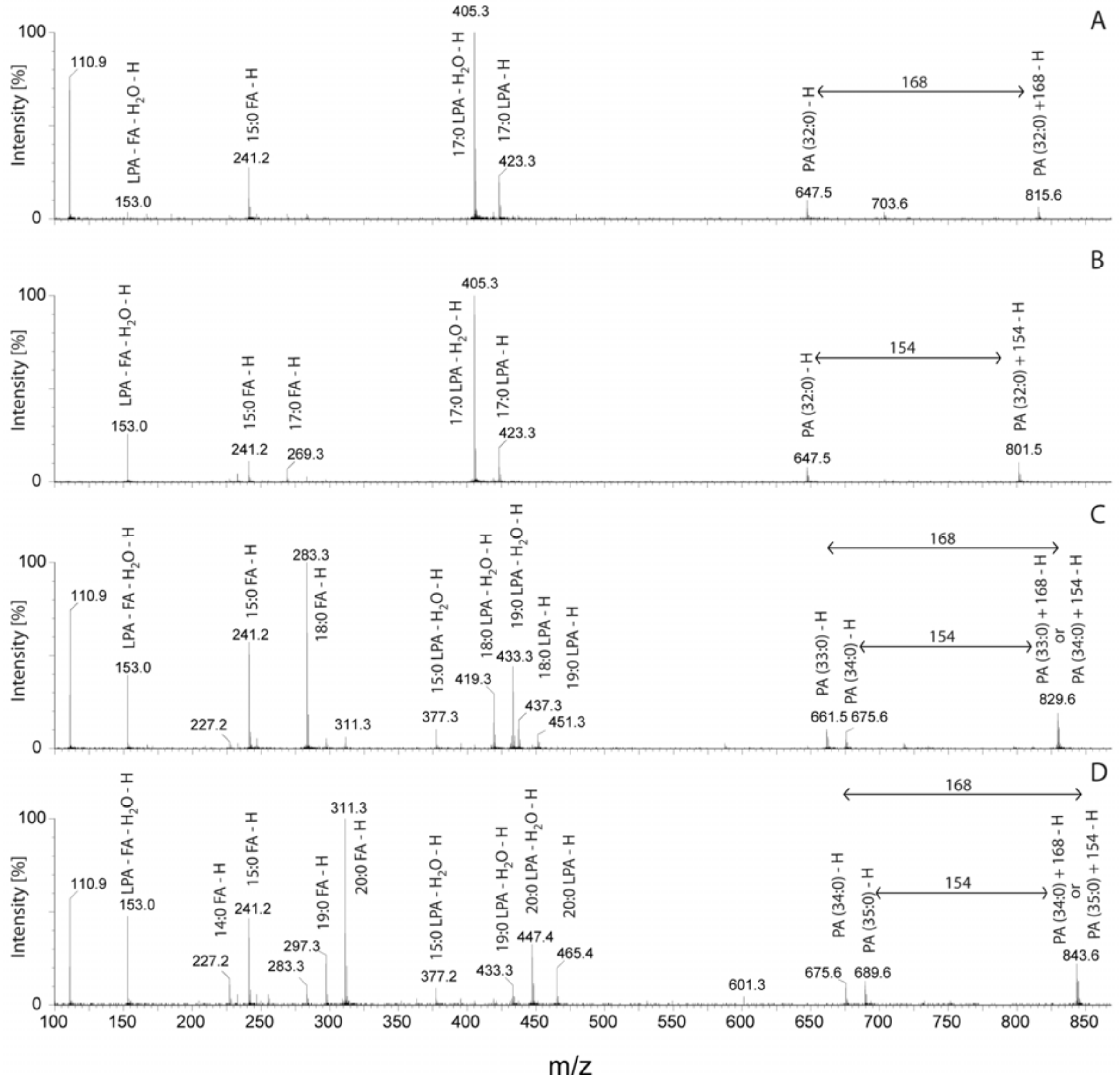

Figure S4: MS-MS-spectra of unidentified compounds Un-3 (B, C, D) and Un-4 (A, C, D) detected in strain P23. Spectra were recorded in ESI negative ion-mode.
